# The Feedback Regulation of PI3K-miR-19a, and MAPK-miR-23b/27b in Endothelial Cells under Shear Stress

**DOI:** 10.3390/molecules18010001

**Published:** 2012-12-20

**Authors:** Jian He, Yulin Li, Xufang Yang, Xu He, Haiying Zhang, Jin He, Lihong Zhang

**Affiliations:** 1 The Key Laboratory of Pathobiology, Ministry of Education, Norman Bethune College of Medicine, Jilin University, Changchun 130021, China; 2 Departments of Bioengineering and Medicine and Institute of Engineering in Medicine, University of California, San Diego, La Jolla, CA 92037, USA; 3 Department of Pathophysiology, Mudanjiang Medical College, Mudanjiang 157000, China; 4 The First Clinical Hospital, Jilin University, Changchun 130021, China

**Keywords:** endothelial cell, feedback loop, mechanical force (shear stress), microRNA, PI3 Kinase/MAP Kinase

## Abstract

Mechanical stimulation regulates endothelial cell (EC) functions through the modulation of signaling networks and gene expression. Our recent studies have identified that shear stress regulation of microRNAs (miRs)-19a, 23b and 27b, led to the modulation of EC proliferation. However, the underlying molecular mechanisms by which shear stress regulates these miRs have not been explored. Previous studies showed that shear stress activates multiple signaling pathways, including phosphatidylinositol 3 kinase (PI3K) and mitogen-activated protein kinase (MAPK). In this work we demonstrate that inhibition of the PI3K pathway attenuated the shear-induced miR-19a, and inhibition of the MAPK pathway attenuated miR-23b, 27b. The knockdown of miR-19a using antagomir-19a oligonucleotide (AM19a) decreased the shear-induced PI3K activation; whereas AM-23b, 27b reduced the shear-induced MAPK activation. Furthermore, the overexpression of miR-19a overrode the suppressive effects of PI3K inhibitors on shear-induced PI3K activation; the overexpression of miR-23b, 27b had similar effects on ERK activations, but had little effect on P38 and JNK activation. Our findings suggest a positive feedback loop whereby PI3K and MAPK mediate the shear regulation of miR expression, which in turn modulates the shear-regulated PI3K/MAPK signaling events in ECs.

## Selected Abbreviations and Acronyms

HUVECs: human umbilical venous endothelial cells
MiRs: microRNAs
PI3K: phosphoinositide 3-kinase
MAPKs: mitogen-activated protein kinases
ERK: extracellular signal-regulated kinase

## 1. Introduction

Hemodynamic forces regulate the structure and function of the blood vessel wall, which was reported by Langille *et al.* [1]. Vascular endothelial cells (ECs), located at the interface between the circulating blood and the blood vessel, are exposed to shear stresses resulting from the tangential forces exerted by the flowing fluid on the vessel wall, leading to the modulation of signaling networks and expression of microRNAs [2,3,4]. ECs respond to changes of blood flow and distending pressure and convert mechanical stimuli into intracellular signals to affect cellular functions, e.g., proliferation, apoptosis, migration, permeability, and remodeling, as well as gene expression [3,5]. In the arterial tree, regional differences in shear stress forces produce distinct effects on the EC phenotype. Laminar shear, present in the straight portions of the tree, elicits a potential anti-inflammatory and atheroprotective response in ECs [6]. In previous studies, we focused on this atheroprotective shear stress force and found an upregulation of a distinct group of miRNAs that led to distinct functional consequences [7,8]. MicroRNAs (miRs) are short noncoding 18–24 nucleotide RNAs that negatively regulate the expression of target genes at the posttranscriptional level [9]. Among the mechano-sensetive miRs in ECs, atheroprotective shear stress induces miR-23b, 27b and 19a leads to EC growth arrest [7,8]. However, the mechanisms by which shear stresses regulate miR expression remain unexplored. 

Previous studies showed that mechanical forces, exerted by fluid shearing, activate the phosphatidylinositol 3 (PI3) kinase and mitogen-activated protein (MAP) kinase pathways [10,11,12,13] and the shear-induced activations can be attenuated by specific chemical inhibitors [14,15,16]. Activation of PI3K and MAPK pathways has been implied to promote EC cell proliferation, migration and survival [10,17,18,19]. The role of miRs in PI3K and MAPK-modulated EC functions under shear remains undetermined. 

In this work we found the inhibition of the PI3K pathway attenuated the shear-induced expression of miR19a, and inhibition of the MAPK pathway attenuated shear-induced miR-23b, 27b. Inhibition of miR-19a using antagomir-19a oligonucleotide (AM19a) diminished the shear-induced PI3K/AKT activation; similarly, inhibition of miR-23b, 27b using antagomir-23b oligonucleotide (AM23b) and antagomir-27b oligonucleotide (AM27b), respectively, reversed the shear-induced MAPK activation. Overexpression of miR-19a using pre-miR-19a significantly attenuated the blockade effects of PI3K inhibitor; likewise, overexpression of miR-23b, 27b significantly attenuated the blockade of MAPK inhibitor. Our findings indicate a feedback loop in which PI3K/AKT and MAPK mediate shear-regulation of miRs expression, and miRs as well modulate PI3K/AKT and MAPK signaling in human ECs under shear conditions.

## 2. Results

### 2.1. Inhibition of PI3K and MAPK Pathways Attenuated the Shear-Induction of miR-19a and miR-23b/27b, Respectively

**Figure 1:**
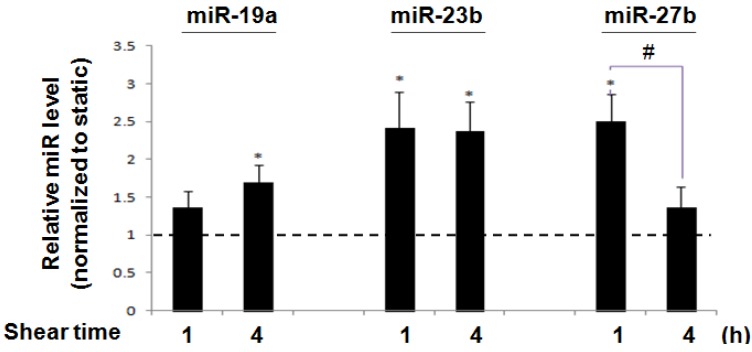
Shear stress regulation of endothelial cells. Laminar shear stress regulated miR expressions. QRT-PCR shows that laminar shear stress (12 dyne/cm^2^) significantly upregulated miR-19a at 4h, miR-23b at 1 h and 4 h, and miR-27b at 1 h. * *p* < 0.05 (compared with 1), # *p* < 0.05 between two time points. Data are mean ± SEM (n = 6).

Using qPCR, we compared the expression of miRs in ECs after exposure to a laminar shear stress of 12 dyne/cm^2^ for the indicated time periods with those cultured under static conditions for the same time periods. MiR-19a was significantly increased at 4 h (1.689 ± 0.238 fold in comparison to the time matched static control) after shearing. MiR-23b was significantly increased as early as 1 h (2.42 ± 0.48 fold) and this lasted at least for 4 h (2.37 ± 0.40 fold); miR-27b was significantly increased (2.50 ± 0.36 fold) at 1 h, and decreased to 1.37 ± 0.27 fold at 4 h (Figure 1). These results demonstrate that they were relatively early-responsive miRs to shear stress in ECs.

**Figure 2:**
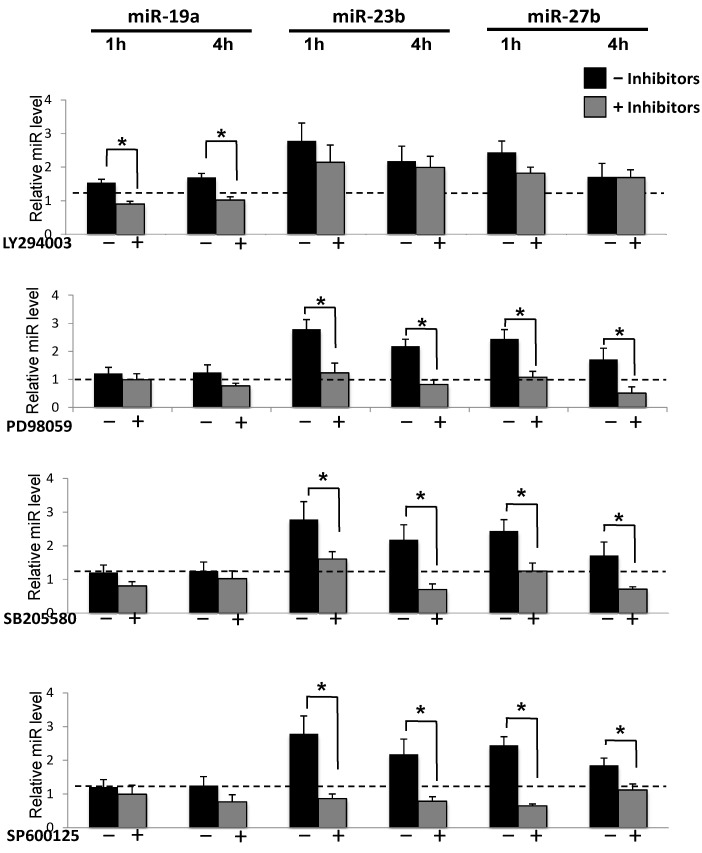
AKT and MAPK in shear-regulation of miRs. 10 µM LY 294002 (PI3K inhibitor), PD 98059 (MAPK inhibitors), SB 203580 (pP38 inhibitor) and SP 600125 (pJNK inhibitor) were used to block the shear-induced phosphorylation of AKT, ERK1/2, p38 and JNK in ECs. 10µM DMSO was employed as control. QRT-PCR shows the inhibition of PI3K pathway attenuated the shear-induced miR-19a, but there were no significantly changes of miR-23b and 27b; inhibition of MAPK pathway attenuated the shear-induced miR-23b and 27b, but not miR-19a. Data are presented as the mean ± SEM (n = 6). * *p* < 0.05 (compared with the static control at the same time point).

It has been shown that shear caused quick activations of PI3K/AKT and all three MAPKs [10,11,12,13]. We proceeded to investigate the roles of PI3K/AKT and MAPKs in shear-induction of miR expression. Under shear, treatments with LY294002 (PI3K inhibitor) and PD98059 (ERK inhibitor), SB203580 (p38 inhibitor) and SP600125 (JNK inhibitor) significantly attenuated the shear-induced phosphorylation of AKT, ERK1/2, P38, and JNK, respectively (Supplementary Figure S1). These results confirmed the inhibitory effects of these inhibitors on the flow-activation of each of their cognate kinases. Moreover, the inhibition of PI3K abolished shear-induced miR-19a expression, but had little effect on miR23b and 27b expression (Figure 2). The inhibition of ERK, JNK, or P38 attenuated the shear-induced miR-23b/27b expression, but not miR19a (Figure 2). These results identify the potential differential signaling pathways for shear-induced miR-19a and miR-23b/27b expressions. 

### 2.2. The Knockdown of miR-19a Decreased the Shear-Induced PI3K Activation; and Knockdown of miR-23b and miR-27b Reduced the Shear-Induced MAPK Activation

**Figure 3:**
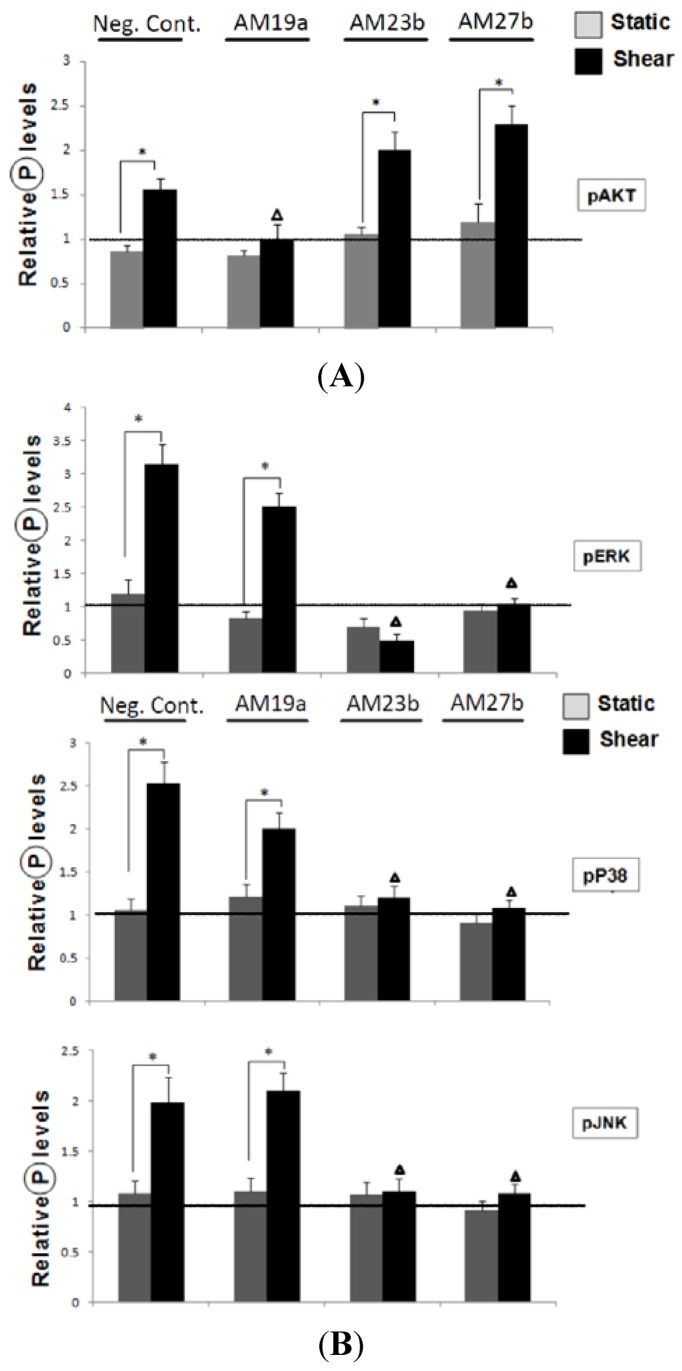
Inhibition of miRs in shear-regulation of AKT and MAPK. (**A**) Antagomir against miR-19a decreased the shear-induced PI3K activation, but had no effect on MAPK activation; (**B**) Antagomir against miR-23b, 27b decreased the shear-induced MAPK activation, but had no effect on PI3K activation. Data are presented as the mean ± SEM (n = 3) (Data were normalized to the control in Neg Cont. group). * *p* < 0.05 (Compared with the Static); in pAKT assay, ^∆^ *p* < 0.05 (Compared with the shear groups in Neg Cont., AM23b and AM27b groups; in pERK, pP38, pJNK assay, ^∆^ *p* < 0.05 (Compared with the shear groups in Neg Cont. and AM19a groups).

In order to investigate the interplay between miR expression and signaling pathways, we performed the loss-of-function analyses to study the effects of miR-19a, 23b and 27b knockdowns on shear-induced AKT and MAPK phosphorylation (Supplementary Figure S2). Antagomir against miR-19a, miR-23b and miR-27b (AM19a, 27b and 23b) were transfected into HUVECs to reduce the endogenous levels of these miRs. The target genes of miR-19a and 23b/27b are cyclin D1 (CCND1) and E2F1, respectively. The increased expression of the target genes indicated the AM transfection worked. Transfection of AM19a significantly decreased the shear-induced AKT activation (Figure 3A) in comparison the cells transfected with negative control AMCs. Transfection of AM23b and AM27b reduced the shear-induced MAPK activation (Figure 3B). These results suggest a feedback loop in which AKT mediated shear-induction of miR-19a, which may lead to a further increase of the shear-induced AKT activation. A similar feedback mechanism is also observed in MAPK-miR-23b/27b pathway.

### 2.3. The Overexpression of miR-19a Attenuated the Suppressive Effects of PI3K Inhibitors on Shear-Induced PI3K/AKT Activation; the Overexpression of miR-23b, 27b Overrode the Inhibitory Effects of PD98059 on ERK Activations

**Figure 4:**
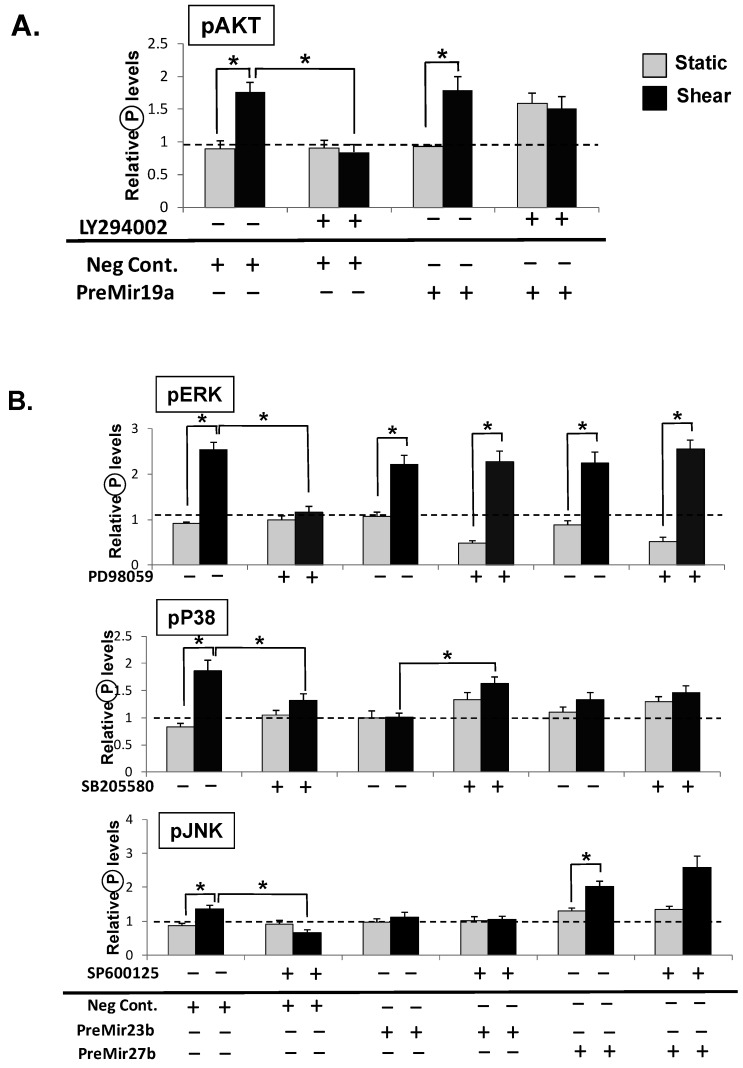
Overexpression of miRs in AKT and MAKP phosphorylation. (**A**) Transfection of miR-19a precursor enhanced AKT phosphorylation; (**B**) Transfection of miR-23b, 27b precursors overrode the suppressive effects of ERK inhibitors on shear-induced ERK, but not p38 and JNK, activation. Pre-MiR-27b overrode the SP600126 inhibition of JNK phosphorylation under shear. Data are presented as the mean ± SEM (n = 3). * *p* < 0.05.

To further establish the correlations between signaling pathways and miR expression under shear, we performed gain-of-functions analyses to investigate the effect of overexpression of miRs in shear-induced signaling. Precursor microRNAs (PreMiRs) were used to increase the expression levels of the corresponding miRs (Supplementary Figure S3). We found that the overexpression of miR-19a overrides the suppressive effects of AKT inhibitors on shear-induced AKT activation (Figure 4A); the overexpression of miR-23b, 27b had similar effects on shear-induced ERK activation (Figure 4B). However, PreMiR23b and 27b did not reverse the inhibitory effects of SB203580. PreMiR27b also overrode the inhibitory effects of SP600125 on shear-induced JNK phosphorylation. Notably, Pre-MiR23b and 27b slightly increased P38 and JNK phosphorylation under both static (ST) and sheared (SS) conditions (Figure 4B). These results demonstrated that miR-19a, miR-23b/27b play important roles in regulating EC signaling. Together with the data of loss-of-function of AM-19a, 23b and 27b in laminar shear-induced PI3K and MAPK pathways, the results suggest a feedback loop of PI3K-miR-19a, and MAPK-miR23b/27b.

## 3. Discussion

MiR-19a consists of mir-17-92 microRNA clusters with miR-17, miR-18, miR-19b, miR-20, miR-25, miR-92, miR-93, miR-106a, and miR-106b [20], which are usually considered as oncogenic miRNA, which promotes cell proliferation and angiogenesis [21,22,23]. The bioinformatics analyses identify that miR-17-92 targets p21 that contributes to cell proliferation: targets PTEN/PI3K/AKT for anti-apoptotic effects; and targets CTGF and Tsp1 to promote angiogenesis [21]. Besides these target genes, it has been recently reported that TNF is one of the target genes in esophageal cancer development. The inhibition of miR-19a leads to esophageal cancer cell growth arrest and apoptosis [24]. However, it has been shown that miR-19a is induced by laminar shear stress to target cyclin D1 and this led to an attenuation of the G1/S transition in ECs [8], indicating the role of miR-19a is beyond oncogenesis and cell cycle angiogenesis.

The PI3K/AKT pathway plays an important role in many cellular functions. Activation of PI3K/AKT pathway signaling leads to the mTOR-mediated tumor angiogenesis [25,26]. In particular, PI3K involves in multiple EC functions, e.g., promoting EC proliferation, migration and survival [26]. Both PI3K and miR-19a has been shown to be involved in EC mechanotransduction [10,27]. The role of the PI3K pathway in regulating miR19a is not examined. Our current study showed that the inhibition of PI3K pathway attenuated the shear-induced miR19a, the knockdown of miR19a using AM19a decreased the shear-induced PI3K activation and the overexpression of miR-19a enhanced the AKT phosphorylation in ECs treated with LY 294002, and attenuated its suppressive effects on shear-induced PI3K activation. These results suggest a possible feedback loop whereby PI3K mediates shear regulation of miR expression.

MiR23b and 27b are members of the MiR-23-27-24 cluster, which is highly expressed in many vascularized tissues, and play critical roles in cardiovascular development, angiogenesis, and EC homeostasis [28]. Recently it is reported that miR-27b controls endothelial tip cell fate, branching, and venous specification by determining Spry2 (Sprouty homologue 2) and Dll4 (Delta-like ligand 4) as its essential targets.

The inhibition of Spry leads to the activation of MAPK according to the literature. This is another piece of strong evidence to demonstrate that miR-27b plays important roles in regulating EC signaling [29]. MiR-23b and 27b were shown to be induced by shear stress [2,7,30], and MiR23b played an important role in shear-induced G2/M EC growth arrest [7]. Shear-induced G2/M arrest and the corresponding changes in G2/M regulatory protein expression through Smad 1/5 have been reported [31]. MiR-23b targeting Smads (Smad3, Smad4 and Smad5) serve as a molecular switch in regulating transforming growth factor-β1 (TGF-β1) in regulating liver stem cell differentiation [32], a model in which miR-23b clusters promote the growth of fetal hepatocytes by down-regulating Smads and consequently TGF-β signaling [30]. MAPK-mediated phosphorylation of Smads 2 and 3 may confer resistance to TGF mediated growth inhibition in cancer cells harboring a hyperactive Ras/MAPK pathway [33]. One report has shown that TGF induces expression of miR23 cluster (miRs-23a, 27a, and 24) in Huh7 cells through a Smads dependent mechanism [34]. Akiko Hata’s [35] group showed that Smad proteins control Drosha-mediated microRNA-21 maturation and Smad proteins bind a conserved RNA sequence to promote microRNA maturation by Drosha [36]. Here, we have shown that the Smad upstream signaling molecules, MAPKs, are involved in the shear-induced miR23b/27b expressions, and miR-23b/27b also played a feedback role in controlling shear-induced MAPK activation. The role of Smads in mediating shear-regulated MAPK-miR loop remains to be determined. In addition, it is interesting that the overexpression of miR23b/27b with PreMiRs, overrode the PD98095 inhibition of shear-induced ERK phosphorylation, which suggests that miR23b/27b may be targeting some negative regulator for ERKs, however the overexpression of miR23b/27b did not resume the shear-inducibility for P38 and JNK after treatment with inhibitors, suggesting that miR23b/27b may play a more important role in ERK signaling. It is also interesting to note that AM23b caused an elevation of ERK phosphorylation under static condition; and PreMiR23b/27b reduced ERK phosphorylation under static condition with PD98095 treatment. These results strongly suggest that miR23b/27b targeting negative regulators for MAPKs. More studies will be needed to elucidate the miR23b/27b targets for MAPK signalings.

In summary, we demonstrated feedback loops that MAPK and PI3K were involved in the regulation of miRs expression in ECs by shear stress. These results revealed a mechanism by which mechanical forces modulate the interplay of miR and mechanotransduction network in ECs.

## 4. Material and Methods

### 4.1. Cell Culture and Reagents

HUVECs were isolated from human umbilical cord veins as previously described [37]. The cells were cultured in Medium 199 (Invitrogen, Carlsbad, CA, USA) supplemented with 10% fetal bovine serum (Omega), 10% endothelial cell growth medium (Cell Applications, San Diego, CA, USA), 1% L-glutamine, 1% sodium pyruvate, and 1% penicillin/streptomycin. All cell cultures were kept in a humidified 5% CO_2_/95% air incubator at 37 °C. Cells within passages 5 to 6 were used in all experiments. The chemical inhibitors LY 294002, PD 98059, SB 203580 and SP 600125 all at the work concentration of 10 µM (EMD Millipore Chemical, San Diego, CA, USA), were used to block the activities of PI3K, ERK, p38 and JNK respectively. Before flow experiments, the cells were subjected 12 h low serum condition (0.5% FBS) followed by the pretreatments with the blocking reagents for 1 h to inhibit the specific kinase functions [15].

### 4.2. Shear Stress Experiments

**Figure 5:**
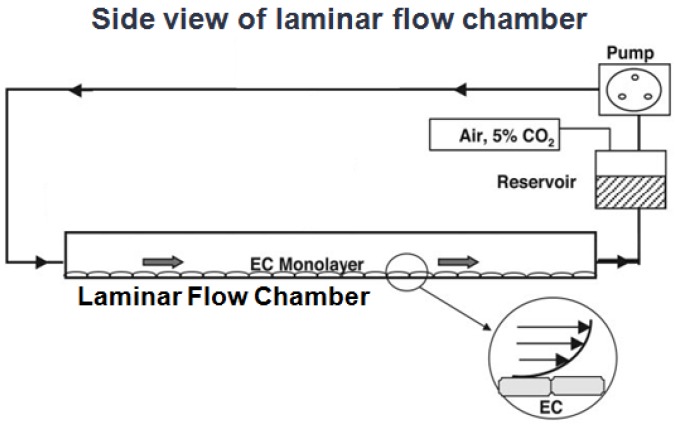
Side view of the laminar flow chamber. The endothelial cell (EC) monolayers cultured to confluence on the bottom surface of the rectangular channel are exposed to the flow generated by a pressure difference between the inlet and the outlet of the chamber. The flow system is maintained at 37 °C in a hood and equilibrated with 5% CO_2_/95% air. The insert in channel shows an enlarged view of two ECs and the flow profile of the applied shear stress.

A parallel-plate flow chamber device was used as an *in vitro* system to study the responses of cultured ECs to shear stresses at the cellular and molecular levels as described before [38]. In brief, HUVECs cultured on 38 X 76-mm slides and assembled into a chamber in that a flow channel was created by using a gasket with a rectangular cutout with uniform channel height along the flow path. HUVECs in the rectangular channel are exposed to laminar shear stress (SS) at 12 dyne/cm^2^ generated by a pressure difference between the inlet and the outlet of the chamber (Figure 5). The flow system was maintained at 37 °C in a hood and equilibrated with 5% CO_2_/95% air.

### 4.3. MicroRNA RT-qPCR

Quantitative assessment of specific miR levels was performed using the TaqMan miR assay kits (Applied Biosystems, Carlsbad, CA, USA) according to the manufacturer’s protocol. MiR cycle threshold (Ct) values were normalized to internal control (RNU48) and converted into copy numbers, by ΔΔCT method [39], and shear groups were then normalized to the time match static controls.

### 4.4. Western Blot Analysis

Western Blot analysis was performed using standard protocols. Antibodies against pAKT, total AKT, pERK1/2, pP38, P38 were obtained from Cell Signaling, and anti-GAPDH, ERK2, pJNK, JNK2 were obtained from Santa Cruz (Santa Cruz, CA, USA). These were used in a 1:1,000 dilution, and an HRP-conjugated secondary antibody, followed by enhanced chemiluminescence detection.

### 4.5. Pre-mir and Antagomir Transfection

Premirs were transfected into ECs to increase the expression levels of the corresponding miRs; antagomirs against miR-19a and 23b/27b, as well as negative control molecule (Anti-miR™ miRNA Inhibitor Negative Control #1), were purchased from Ambion (Carlsbad, CA, USA). The oligonucleotides were individually transfected with siPort NeoFx reagent (Ambion, Carlsbad, CA, USA) at the final concentration of 50 nM, 12-h post-transfection. Using qPCR to detect the expression of the target genes of miR-19a and 23b/27b, CCND1 and E2F1resepctively after transfected 12 h. The increased expression of the target genes indicated the transfection of AM worked, and the decreased expression of the target genes meant the Premirs transfection worked.

## 5. Conclusions

Our findings suggest a positive feedback loop in which PI3K and MAPK mediate shear regulation of miR expression, which in turn modulates the shear-regulated PI3K/MAPK signaling events in ECs.

## Supplementary Materials

Supplementary materials can be accessed at: http://www.mdpi.com/1420-3049/18/1/1/s1.
