# Supplementary material for: The Feedback Regulation of PI3K-miR-19a, and MAPK-miR-23b/27b in Endothelial Cells under Shear Stress
Source: Molecules. 2012 Dec 20;18(1):1–13. doi: 10.3390/molecules18010001 (PMC6269661; doi:10.3390/molecules18010001)

Supporting Information

Figure S1. 10 μM LY 294002 (pAKT inhibitor), PD 98059 (MAPK inhibitors), SB 203580 (pP38 inhibitor) and SP 600125 (pJNK inhibitor) blocked the shear-induced phosphorylation levels for AKT, ERK1/2, p38 and JNK in ECs. 10 μM DMSO was employed as control. Data are presented as the mean ± SEM (n = 3, compared with static control), * $p < 0.05$ (compared with 1).

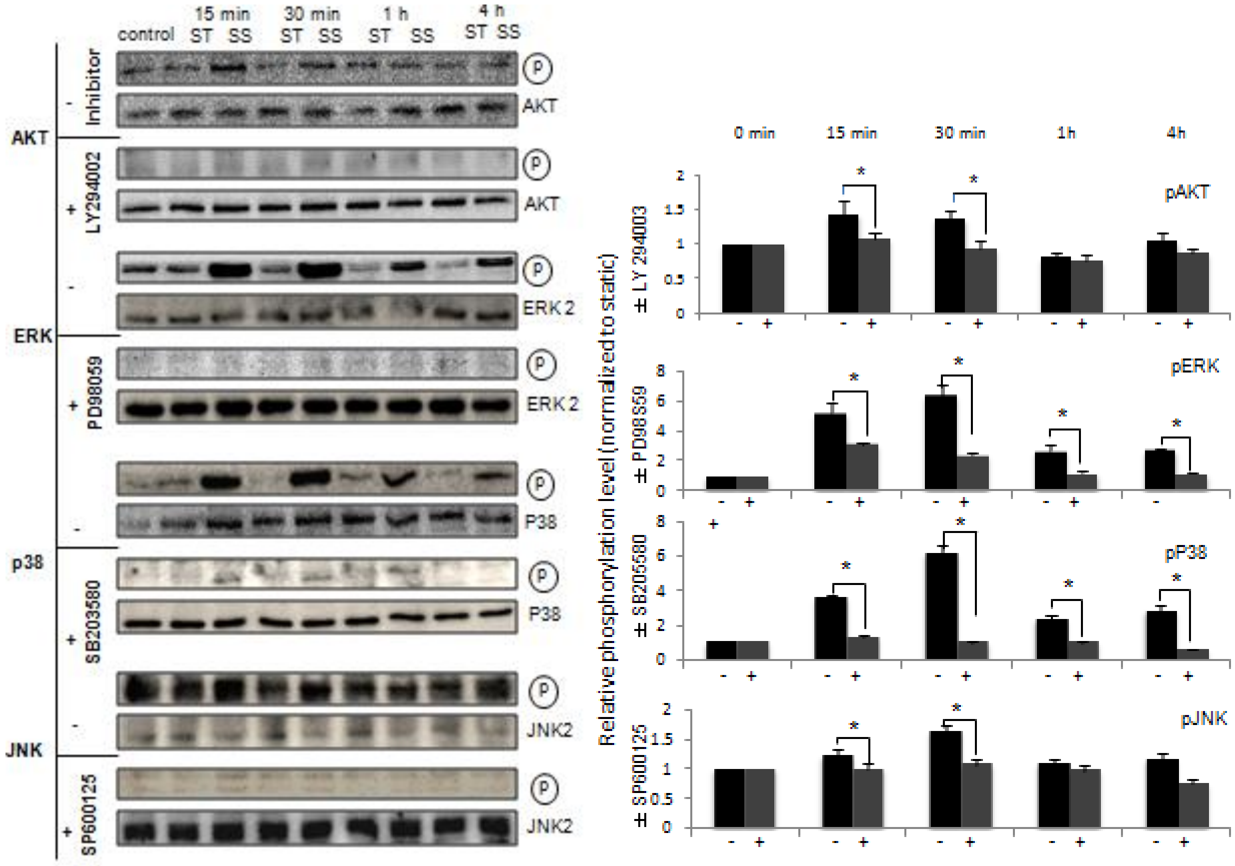

Figure S2. Antagomir against miR-19a decreased the shear-induced PI3K activation; Antagomir against miR-23b, 27b decreased the shear-induced MAPK activation.

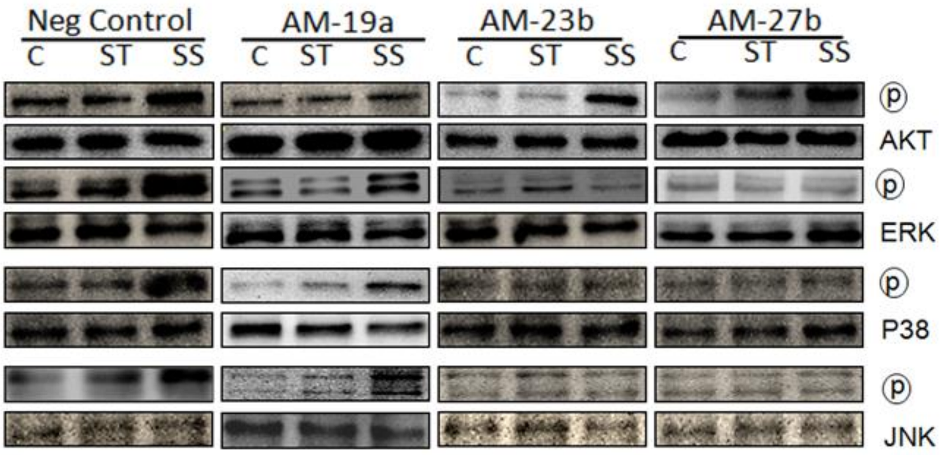

Figure S3. Over expression of miR-19a overrode the suppressive effects of PI3K inhibitor on shear-induced PI3K activation (upper). Over expression of miR-23b, 27b overrode the suppressive effects of ERK inhibitors on shear-induced ERK activation. Pre-MiR23b and 27b increased P38 phosphorylation at all time 0 points under, static and sheared conditions (lower).

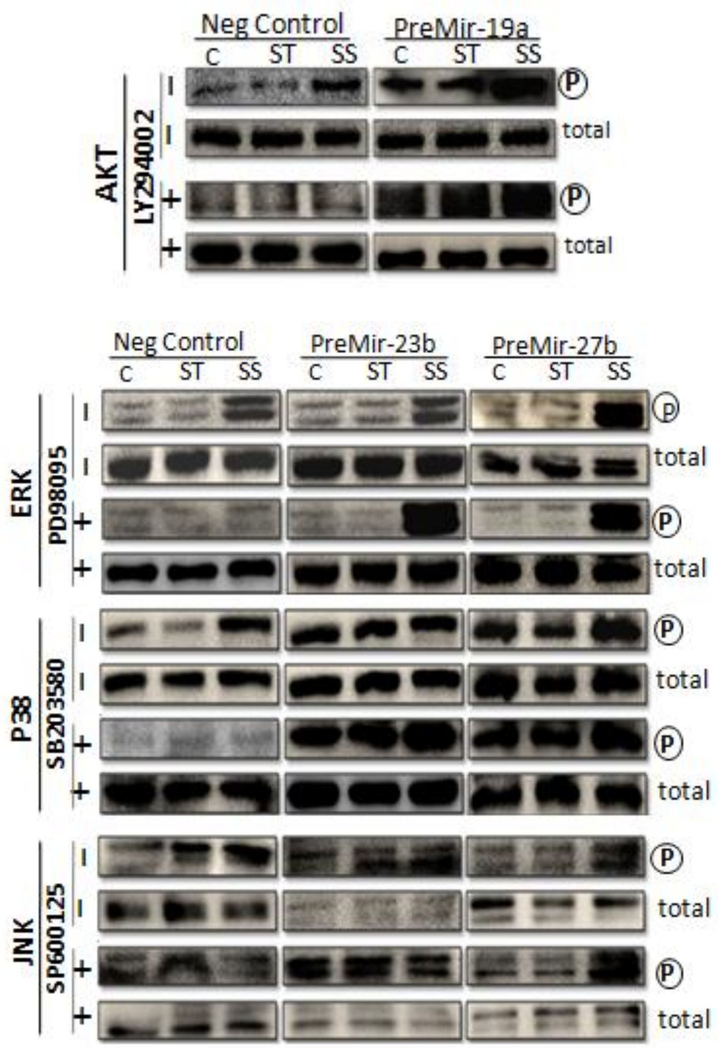

Supplement: Supplementary file 1 [file molecules-18-00001-s001.pdf]
